# Surviving the Deadly Triad: Two Cases of Austrian Syndrome in Southern Switzerland, Associated With Disseminated Streptococcus pneumoniae Infection

**DOI:** 10.7759/cureus.78635

**Published:** 2025-02-06

**Authors:** Valentina Guerini Giusteri, Marco Bongiovanni, Enos Bernasconi, Marco Pons

**Affiliations:** 1 Department of Internal Medicine, Ente Ospedaliero Cantonale, Lugano, CHE; 2 Division of Infectious Diseases, Ente Ospedaliero Cantonale, Lugano, CHE; 3 Faculty of Biomedical Sciences, University of Southern Switzerland, Lugano, CHE; 4 Department of Internal Medicine, University of Geneva, Geneva, CHE

**Keywords:** aortic valve endocarditis, austrian syndrome, invasive pneumococcal disease, septic arthritis, streptococcus pneumoniae

## Abstract

Austrian syndrome is a rare and life-threatening condition. It is more severe in immunocompromised patients, those with comorbidities, or harmful alcohol consumption. The etiopathogenesis involves hematogenous seeding of *Streptococcus pneumoniae* and local spread in the infected tissues, presenting with pneumonia, endocarditis, and meningitis. A 68-year-old man was hospitalized for impaired consciousness, left hemiparesis, and aphasia, consistent with meningoencephalitis. A CT scan showed otomastoiditis and bilateral pulmonary consolidations with respiratory failure. A transesophageal echocardiogram revealed endocardial vegetations at the mitral-aortic junction and left ventricular outflow tract, requiring aortic valve replacement. He also developed septic arthritis in both prosthetic hips and bilateral muscular abscesses. The second patient is a 50-year-old man who was hospitalized for lumbar back pain, aphasia, left-sided neglect, and a motor deficit in the right lower limb. A CT scan showed spondylodiscitis at the L5-S1 level, an abscess in the right frontal region (both confirmed on MRI), and bilateral pulmonary consolidations. A transesophageal echocardiogram revealed endocardial vegetation on the aortic valve. In both patients, *S. pneumoniae* susceptible to penicillin was isolated from all septic sites. After the initial treatment with empirical broad-spectrum antibiotics, therapy was switched to intravenous ceftriaxone and then oral amoxicillin. From these cases, it appears that Austrian syndrome usually requires an intensive approach, combining both medical and surgical interventions to guarantee a positive outcome. It is mandatory to identify individuals at risk to promote the role of vaccination in preventing the development of Austrian syndrome and, more generally, invasive pneumococcal disease.

## Introduction

Austrian syndrome is a condition that involves the triad of meningitis, pneumonia, and endocarditis, caused by *Streptococcus pneumoniae*. It is a life-threatening clinical condition, and early diagnosis can be challenging. In fact, it carries a high mortality rate, ranging from 20% to 40%, despite aggressive combined medical and surgical management. The exact incidence is not well established, as it depends on various factors such as geographic location, serotype prevalence, and the patient’s comorbidities [1]. The condition has become rarer due to early treatment with penicillin and other beta-lactam antibiotics, as well as the introduction of pneumococcal vaccines [2]. It is classified as a pneumococcal invasive disease (IPD), which consists of the development of pneumococcal infection in normally sterile sites. It tends to be more severe in vulnerable individuals, such as immunocompromised patients, persons with harmful alcohol consumption, and those with multiple comorbidities, such as chronic lung diseases, heart failure, and diabetes mellitus [3]. The main mechanism of occurrence of this condition is thought to be through hematogenous seeding and local spread. However, the development of meningitis, due to pneumococcal crossing of the blood-brain barrier after bloodstream invasion, may be enabled by the release of inflammatory mediators [4].

This report describes two cases of Austrian syndrome that occurred in Southern Switzerland in 2024, characterized by multiple complications and septic emboli, but with a favorable outcome despite prolonged hospitalization.

## Case presentation

Case 1

**Figure 1:**
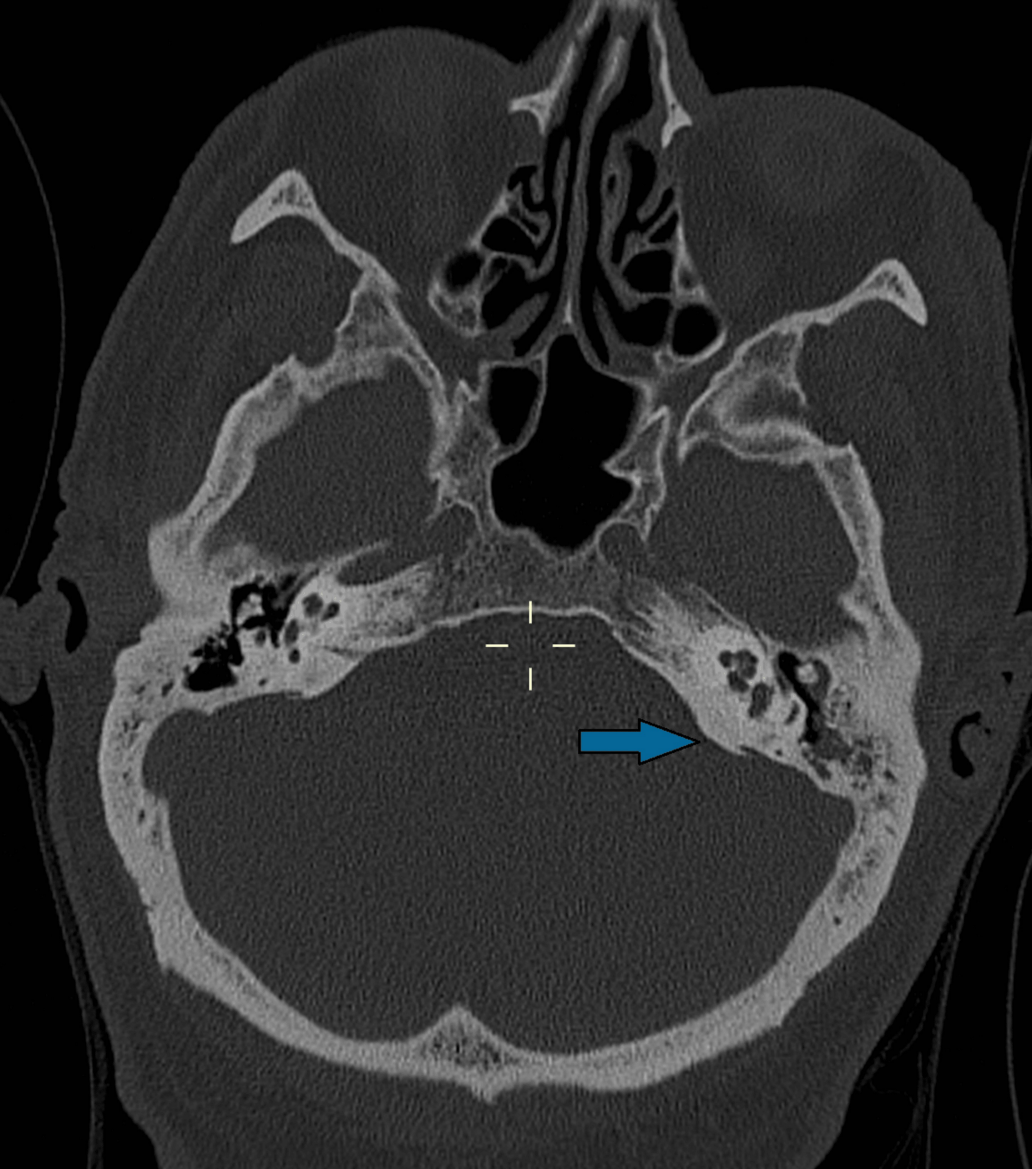
CT scan showing left otomastoiditis.

A 68-year-old man was hospitalized with progressively impaired consciousness, left hemiparesis, and aphasia, which began two days prior. His medical history included permanent atrial fibrillation, third-degree atrioventricular block requiring the implantation of a Micra® pacemaker, moderate aortic stenosis, non-ischemic heart failure with mildly reduced ejection fraction, type 2 diabetes, hypertension, obstructive sleep apnea syndrome with nocturnal Continuous Positive Airway Pressure (CPAP), bilateral hip and left knee replacements, and alcohol consumption (4-6 alcohol units per day). His therapy included a direct oral anticoagulant (rivaroxaban), antihypertensive drugs (valsartan, hydrochlorothiazide, amlodipine, torsemide), and oral hypoglycemic medications (metformin and sitagliptin). The patient's Glasgow Coma Scale (GCS) deteriorated to 8 (E3 V1 M4, eye-opening to verbal command, no verbal response, motor response withdraws to pain), requiring orotracheal intubation, and he experienced a concomitant epileptic seizure, for which diazepam (dosage: 10 mg intravenous, administered only once) and levetiracetam (dosage: 1500 mg every 12 hours) were administered. A brain CT scan ruled out hydrocephalus and showed no signs of hemorrhage or ischemia. However, a chest CT scan revealed bilateral pulmonary consolidations. Given the suspicion of meningoencephalitis, due to turbid CSF observed during lumbar puncture (with >1000 leukocytes/µl, predominance of polymorphonuclear cells, glucose 4.9 mmol/l, proteins 1123 mg/dl), empirical therapy with dexamethasone (dosage: 10 mg every 6 hours), ceftriaxone (dosage: 2 g every 12 hours), ampicillin (dosage: 2 g every 4 hours), and linezolid (dosage: 600 mg every 12 hours) was initiated. *S. pneumoniae* susceptible to penicillin was isolated from both CSF and bronchial aspirate. Treatment was therefore continued with dexamethasone and ceftriaxone. Due to the onset of septic shock, a total body CT scan documented otomastoiditis, identifying the primary source of infection (Figure 1).

**Figure 2:**
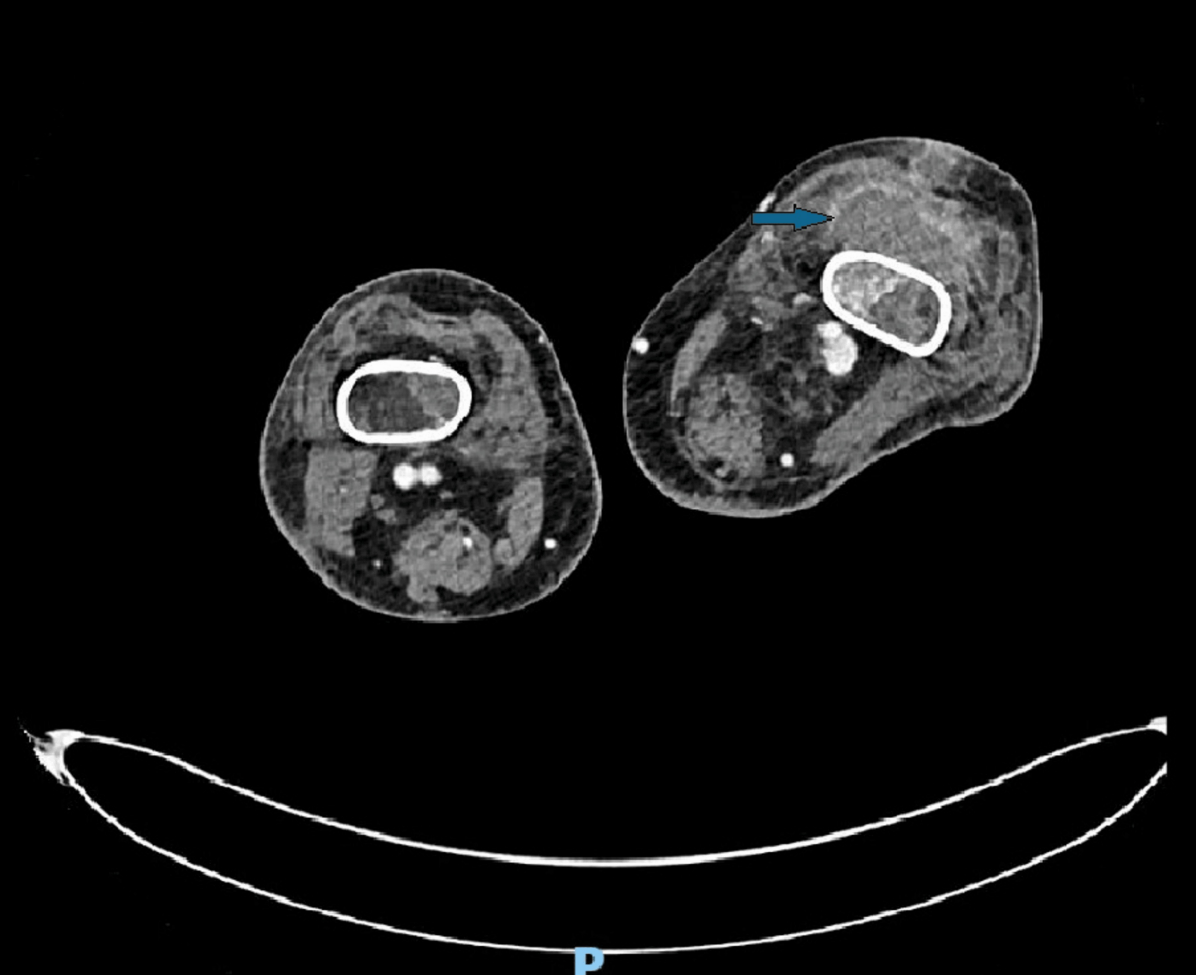
CT scan showing one of multiple muscular abscesses.

A few days later, the patient developed edema of the left lower limb and of the left knee, along with signs of infection in both hips. A CT scan showed bilateral muscular abscesses (Figure 2): one in the right thigh and five in the left limb (sites: left anterior tibial muscle, left posterior tibial muscle, gastrocnemius and soleus, long flexor of the toes extended to the dorsum of the left foot, and the distal third of the left thigh to the dorsomedial subcutaneous fat of the left calf).

**Figure 3:**
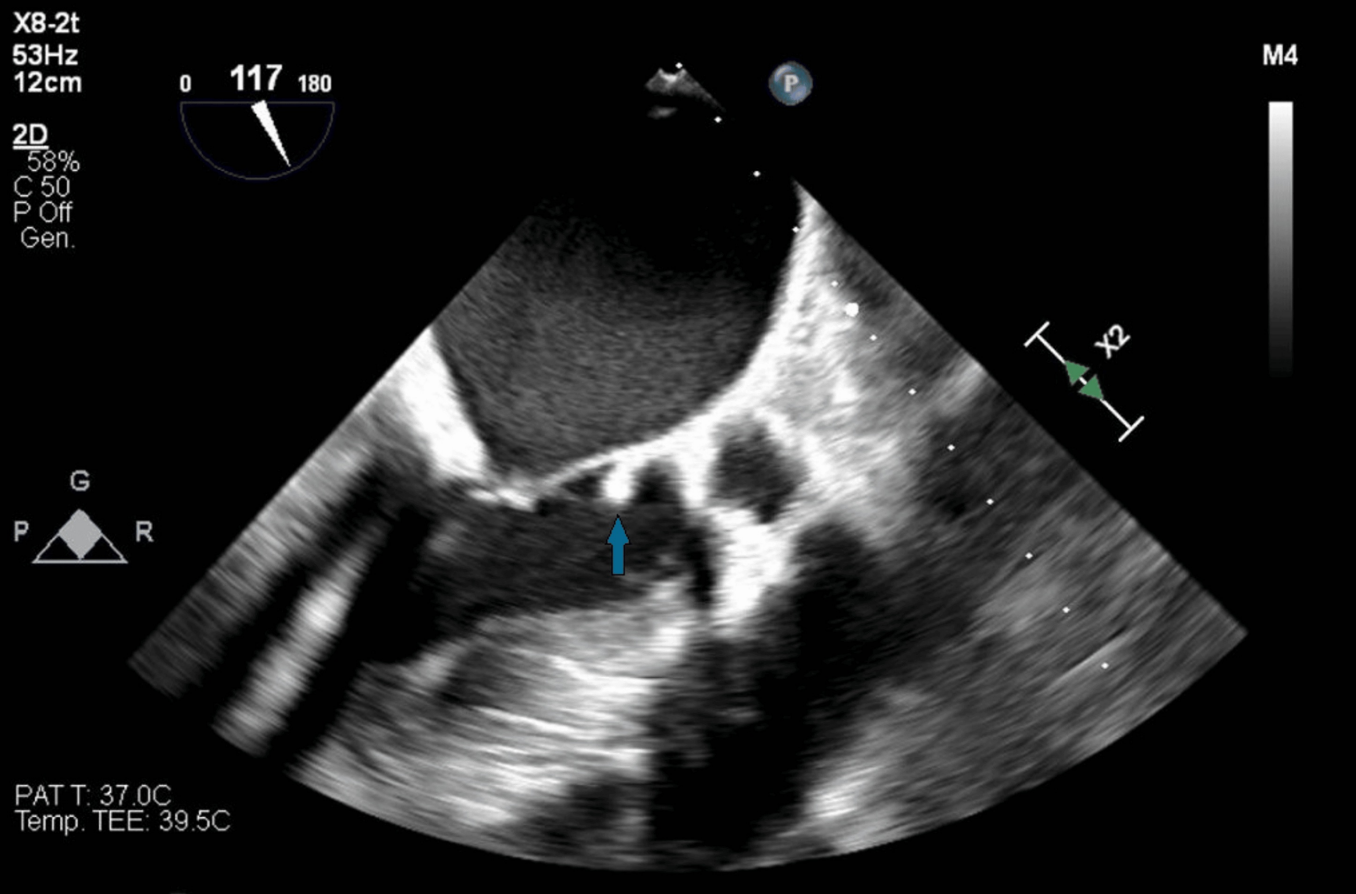
Transesophageal echocardiogram showing vegetation at the mitral-aortic junction.

The spectrum of the antibiotic therapy was empirically extended with meropenem (dosage: 2 g every 8 hours) and vancomycin (loading dose 1500 mg, then 1250 mg every 12 hours) before the 16S ribosomal polymerase chain reaction from diagnostic knee arthrocentesis confirmed the presence of the same *S. pneumoniae*. Intraoperative samples from both hips and the left knee also revealed *S. pneumoniae*, confirming the diagnosis of septic arthritis in the prosthetic joints. Due to the high operative risk associated with prosthesis replacement, the patient underwent debridement surgery, which was repeated twice due to the recurrence of infection at the same sites. A transesophageal echocardiogram was also performed, revealing endocardial vegetations at the mitral-aortic junction and left ventricular outflow tract (Figure 3), with moderate aortic insufficiency, thus confirming Austrian syndrome.

Hence, given the absence of neurological contraindications on brain MRI, the patient underwent aortic valve replacement without complications. During the prolonged hospitalization, the patient developed several complications including critical illness polyneuropathy, fluctuating neurological status, and delirium, managed with quetiapine, all complicating the gradual weaning from mechanical ventilation, requiring a temporary percutaneous tracheostomy. The patient's condition gradually improved, and the antibiotic regimen was switched to oral amoxicillin (at a dosage of 1 g every 8 hours), to be continued in the long term. The patient was finally discharged to a rehabilitation center after two months of hospitalization.

Case 2

**Figure 4:**
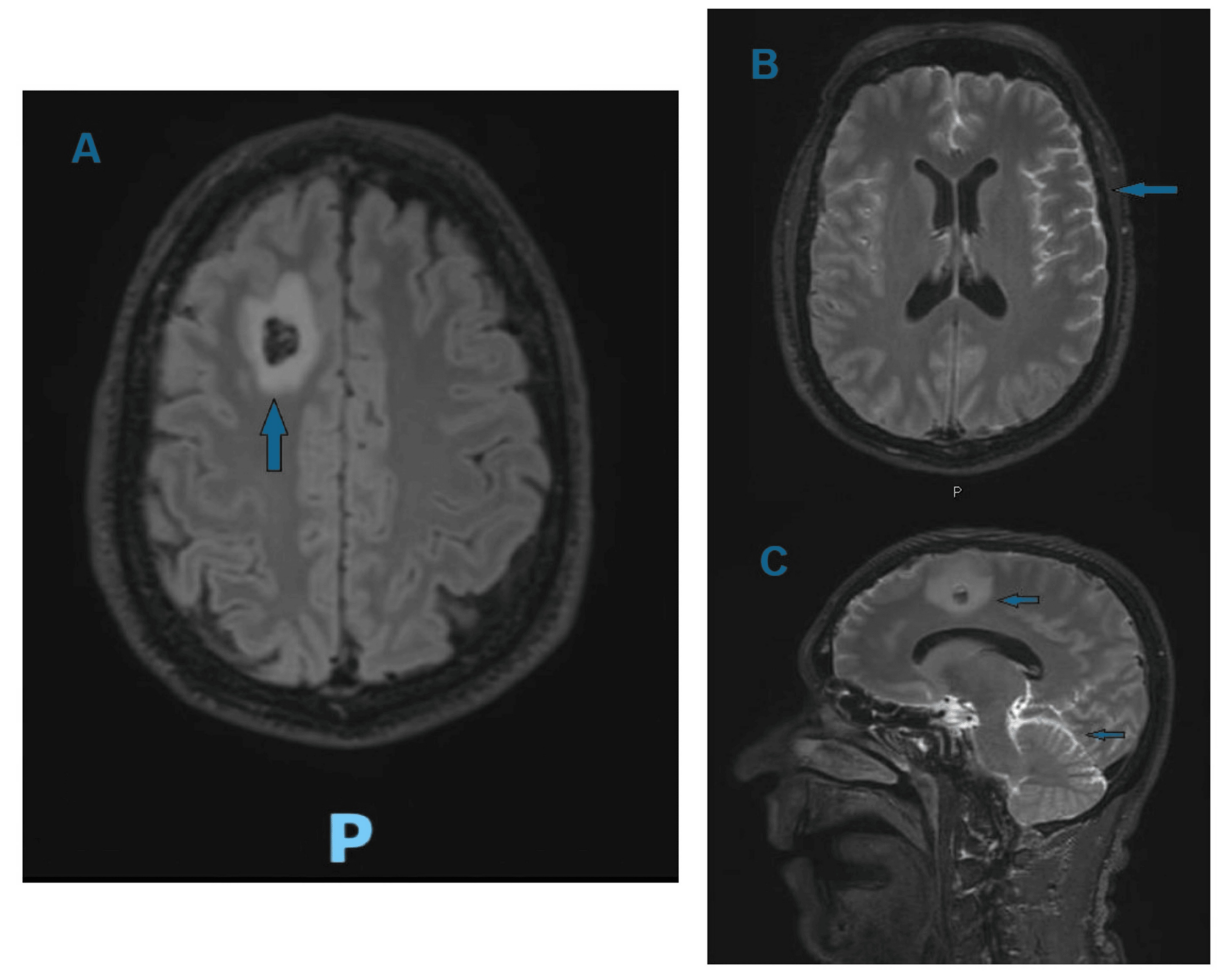
T2-weighted sequence MRI showing an abscess in the right frontal region (A and C) and leptomeningeal enhancement (B and C).

A 50-year-old man with no notable medical history was hospitalized for lumbar back pain, three syncopal episodes, and fever. All symptoms began the day before, and he had taken 600 mg of ibuprofen for pain relief. The patient’s GCS rapidly deteriorated over the day to 10 (E4 V1 M5, meaning spontaneous eye opening, no verbal response, motor response makes purposeful movement to noxious stimuli), associated with vomiting, aphasia, left-sided neglect, and motor deficit in the right lower limb. Suspecting a spondylodiscitis rapidly complicating with the onset of septic shock, an empirical treatment with cefepime (dosage: 2 g every 8 hours) and vancomycin (loading dose 1750 mg, then 1000 mg every 12 hours) was initiated. A brain CT scan revealed a hyperdense area in the right frontal region with surrounding edema, and a lumbar puncture showed turbid cerebrospinal fluid (with 155 leukocytes/µl, predominance of polymorphonuclear cells, glucose 0.2 mmol/l); *S. pneumoniae* susceptible to penicillin (serotype 15C) was isolated from both CSF and blood cultures, so the antibiotic treatment was continued with ceftriaxone (dosage: 2 g every 12 hours). An electroencephalogram ruled out an epileptic seizure. A brain MRI documented the suspected diagnosis of septic embolism and showed diffuse pathological leptomeningeal enhancement, confirming the diagnosis of meningoencephalitis (Figure 4).

**Figure 5:**
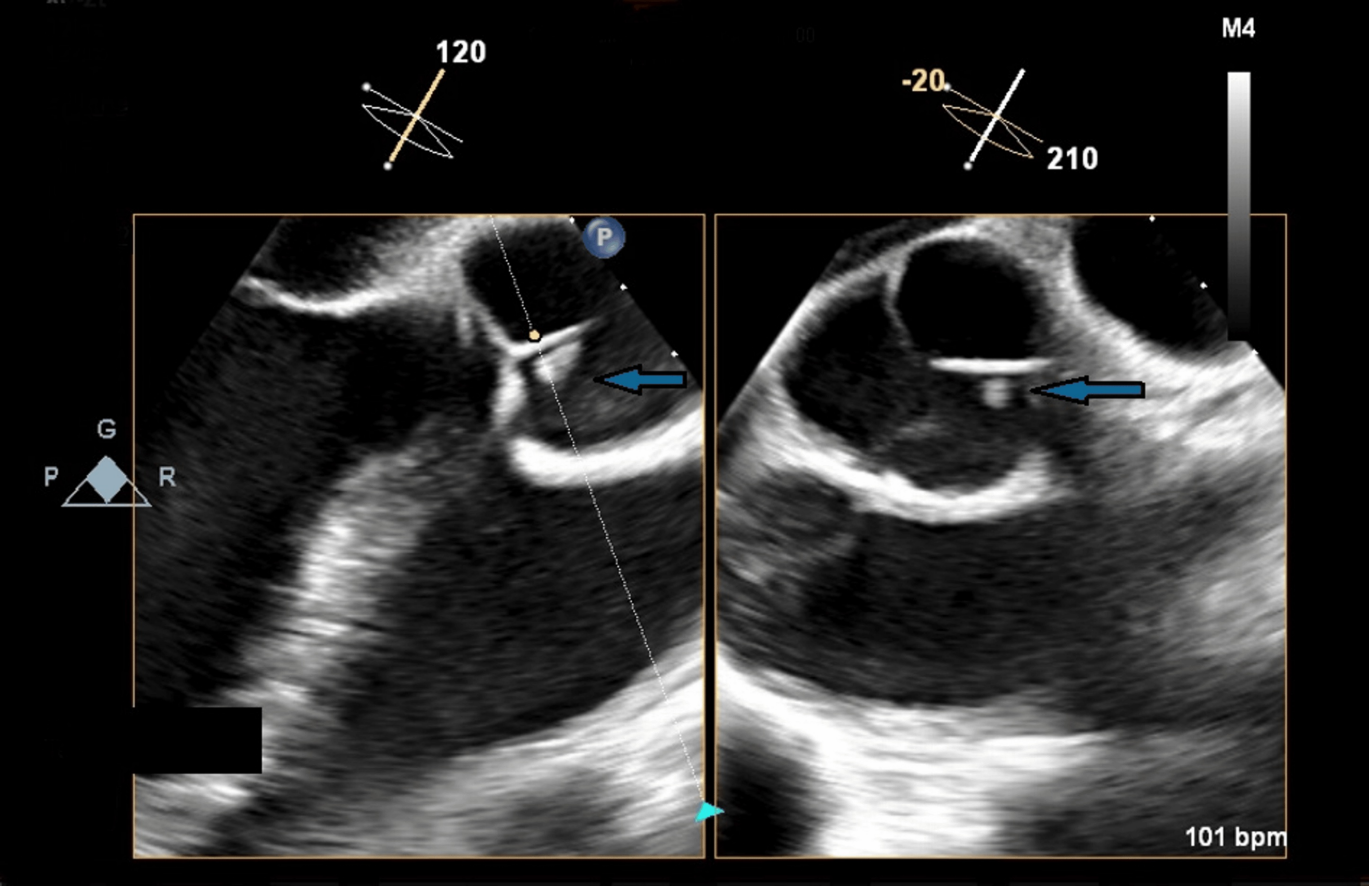
Transesophageal echocardiogram showing vegetation on the aortic valve.

A lumbar MRI was carried out, indicating enhancement of the nerve roots of the cauda equina as well as spondylodiscitis at the L5-S1 level. To establish other sites of septic localization and given the presence of a cough, a thoracoabdominal CT scan showed bilateral pulmonary consolidations due to the same pathogen. A transesophageal echocardiogram was also performed, finding endocardial vegetation on the aortic valve, confirming Austrian syndrome (Figure 5).

The patient also presented several complications. He developed bilateral segmental and subsegmental pulmonary thromboembolism without hemodynamic instability, as well as bilateral superficial thrombosis in arms and legs. These conditions required oral anticoagulation with dabigatran. The patient’s conditions gradually improved, and he was discharged after one month of hospitalization. The antibiotic treatment was switched to oral amoxicillin after three weeks of intravenous ceftriaxone (dosage: 2 g every 12 hours) and continued for a total of six weeks.

**Figure 6:**
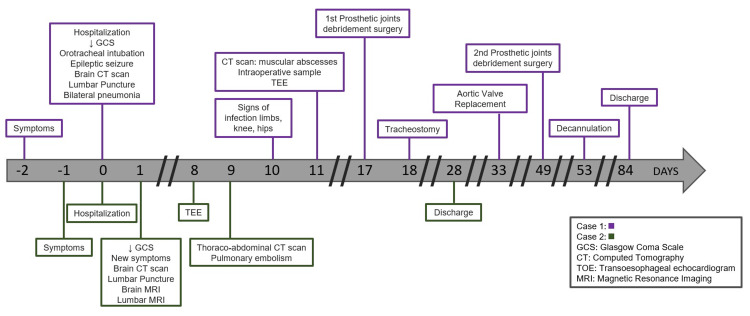
Timeline of events for both cases.

The timeline of events for both cases is reported in Figure 6.

## Discussion

First described in the 19th century, Austrian syndrome is characterized by the triad of pneumonia, endocarditis, and meningitis, following a pneumococcal infection. *S. pneumoniae* is a common cause of otitis, sinusitis, and community-acquired pneumonia [4], which usually have a favorable outcome with effective antibiotic treatment. Nevertheless, several factors increase the risk of developing invasive pneumococcal disease, such as advanced age, male sex, harmful alcohol consumption, previous chronic diseases, and prosthetic joints [3]. One of our patients presents all of these risk factors, particularly alcohol use disorder and chronic conditions such as diabetes, obstructive sleep apnea syndrome, hypertension, and atrial fibrillation.

In addition, our patients were not vaccinated against *S. pneumoniae*, which could have contributed to the aggressive course of the infection. Many studies have highlighted the key role of immunization in preventing invasive pneumococcal disease [2,5]. The CAPITA trial, involving 84,496 adults over 65 years, demonstrated the efficacy of the 13-valent pneumococcal conjugate vaccine (PCV13) in preventing the first episode of invasive pneumococcal disease [6]. Studies on the 23-valent pneumococcal polysaccharide vaccine (PPV23) found similar results, although its effectiveness appears to be age-dependent [7]. Analogous findings were observed with the 20-valent pneumococcal conjugate vaccine [8].

The typical order of appearance of symptoms and localizations of Austrian syndrome is not certain or well-defined. It is proposed that the primary manifestation and portal of entry is pneumonia, usually bilateral. Meningitis is often recognized later, as neurological symptoms may be initially attributed to delirium, and this can influence the neurological prognosis [3,9]. In our patients, neurological alterations such as progressively impaired consciousness, left hemiparesis, and aphasia were the first signs of the disease, leading to early meningitis treatment. Most patients develop severe impairment of consciousness, with a GCS ≤ 8, as well as respiratory failure requiring invasive ventilation [1]. These situations were present in our cases, confirming the aggressive course of Austrian syndrome.

Typically, pneumococcal endocarditis involves left-sided valves (especially the aortic valve), and patients do not develop vasculitic signs. An early surgical approach is essential to prevent cardiogenic shock, as only 17% of cardiac vegetations regress with antibiotic therapy alone [9-12]. In our first case, there was a three-week delay before performing the valve replacement due to the patient’s conditions. In the second case, cardiac surgery was not performed due to the absence of valvulopathy; nonetheless, the prognosis was not affected.

Septic arthritis in Austrian syndrome is uncommon but can be a complication of infective endocarditis, with knee involvement being the most frequent [13]. In our first patient, both hips and the left knee were affected, while the second one developed spondylodiscitis, underscoring the severity of both cases.

In the matter of antibiotic treatment, even though the choice depends on the sensitivity results, a third-generation cephalosporin is frequently used, after an initial period of empirical broad-spectrum antibiotic therapy [1,14,15]. We adopted the same pathway, ending with ceftriaxone and finally amoxicillin.

Vaccination plays a fundamental role in preventing invasive pneumococcal infections. Healthcare strategies must be implemented at local and global levels to identify at-risk individuals and promote vaccination in these sub-groups.

## Conclusions

Several valuable key points can be highlighted from our case. Firstly, it is essential to suspect Austrian syndrome in patients with a pneumococcal infection who also present other risk factors. Secondly, there is a high chance of rapid dissemination, even to foci apart from the three typical localizations, which must be promptly recognized. Thirdly, early diagnosis allows for a more aggressive approach in the context of an extremely serious illness. Therefore, rapid, combined medical and surgical treatment is crucial to offer the patient a better chance of recovery. Finally, it is mandatory to strengthen the role of immunization in preventing the development of Austrian syndrome and, more generally, invasive pneumococcal disease.
